# Comprehensive assessment for miRNA polymorphisms in hepatocellular cancer risk: a systematic review and meta-analysis

**DOI:** 10.1042/BSR20180712

**Published:** 2018-09-25

**Authors:** Ben-Gang Wang, Li-Yue Jiang, Qian Xu

**Affiliations:** 1 Department 1 of General Surgery, The First Hospital of China Medical University, Shenyang 110001, Liaoning Province, China; 2 The Institute of General Surgery, The First Hospital of China Medical University, Shenyang 110001, Liaoning Province, China; 3 The Clinical Medicine, The Fourth Military Medical University, Xi’an 710000, Shanxi Province, China

**Keywords:** hepatocellular cancer, miRNA, meta-analysis, single nucleotide polymorphism, system review

## Abstract

MiRNA polymorphisms had potential to be biomarkers for hepatocellular cancer (HCC) susceptibility. Recently, miRNA single nucleotide polymorphisms (SNPs) were reported to be associated with HCC risk, but the results were inconsistent. We performed a systematic review with a meta-analysis for the association of miRNA SNPs with HCC risk. Thirty-seven studies were included with a total of 11821 HCC patients and 15359 controls in this meta-analysis. We found hsa-*mir-146a* rs2910164 was associated with a decreased HCC risk in the recessive model (*P*=0.017, OR = 0.90, 95% confidence interval (CI) = 0.83–0.98). While hsa-*mir-34b/c* rs4938723 was related with an increased HCC risk in the co-dominant model (*P*=0.016, odds ratio (OR) = 1.19, 95%CI = 1.03–1.37). When analyzing the Hepatitis B virus (HBV)-related HCC risk, hsa-*mir-196a-2* rs11614913 was associated with a decreased HBV-related HCC risk in the co-dominant and allelic models. And hsa-*mir-149* rs2292832 was found to be associated with a decreased HBV-related HCC risk in the dominant and recessive models. In conclusion, hsa-*mir-146a* rs2910164 and hsa-*mir-34b/c* rs4938723 could be biomarkers for the HCC risk while hsa-*mir-196a-2* rs11614913 and hsa-*mir-149* rs2292832 had potential to be biomarkers for HBV-related HCC risk.

## Introduction

MiRNAs are 19–24 nts short nucleotide sequences, which could complementarily combine with multiple target sequences and one miRNA could regulate multiple different target genes [1]. Single nucleotide polymorphisms (SNPs) are the common variations in the genetic polymorphisms and are known as the potential biomarkers for predicting the cancer risk [2]. If there is a variation in miRNA gene, it could affect the quality and quantity of mature miRNA and even affect hundreds of targetted genes regulated by the changed miRNA [3]. There are two types of miRNA-SNP: pri-miRNA SNPs and pre-miRNA SNPs. pri-miRNA SNPs are located over approximately 500–3000bp of the miRNA gene, while pre-miRNA SNPs are found in a 60–70bp region. The function of miRNA-SNPs depends on its location; therefore, pri-miRNA SNPs may have more important roles than pre-miRNA SNPs.

Hepatocellular cancer (HCC) is now the second leading cause of cancer deaths worldwide [4]. In HCC patients, approximately 50% are related with Hepatitis B virus (HBV) [5,6], and HBV is still the major cause of HCC, especially in Asia-Pacific and Sub-Saharan Africa [7]. The etiology of HBV-related HCC is reported different from that of no chronic HBV infection, which is mainly caused by the HBV, host-related such as SNPs, and the dietary and lifestyle factors [8]. Thus, the prediction for the HCC risk, especially the HBV-related HCC risk is essential to prevent the incidence of HCC and increase the early diagnosis of HCC.

Until now, several miRNA-SNPs have been reported to be associated with many tumors such as gastric cancer [9], esophageal cancer [10], breast cancer [11], and neuroblastoma [12]. And miRNA-SNPs were also related with HCC risk [13,14] and could be biomarkers for the precaution for HCC risk, but system analysis or update meta-analysis for all the miRNA-SNPs associated with HCC risk was rare, especially the latest research progress. In addition, many studies supplied data about the HBV-related HCC risk, but few meta-analyses considered this important factor with the etiology of HCC incidence. In the present study, we systematically reviewed published data and comprehensively analyzed and integrated all individual studies for miRNA-SNPs and HCC and/or HBV-related HCC risk. On the basis of systematic review, we conducted a meta-analysis to combine all the available studies and to investigate for the five highly studied miRNA-SNPs whether miRNA polymorphisms contribute to the risk of HCC and/or HBV-related HCC risk.

## Methods

### Publication search

The present study was carried out on the basis of Preferred Reporting Items for Systematic Reviews and Meta-analysis (PRISMA) [15]. Studies reporting on the association between the miRNAs polymorphism and HCC risk were identified by entering the following search terms into PubMed and Web of Science: ‘miRNA’; and ‘polymorphisms/variants/variation/single nucleotide polymorphism/SNPs’; ‘hepatocellular’; and ‘cancer/carcinoma/tumor/neoplasm’ published until 23 February 2018. Two independent investigators (B.-g.W. and Q.X.) performed this literature search. Eligible studies met the following criteria: (i) investigate the relationship between miRNA-SNPs and HCC risk and (ii) case–control study. Articles were excluded based on the following criteria: (i) duplicated articles or data; (ii) not relevant to HCC risk or miRNA-SNPs; (iii) functional studies; and (iv) lack of available data.

### Data extraction

Two investigators (B.-g.W. and Q.X.) extracted the data independently and reached consensus regarding all the items. Study descriptions were derived from the full text including the author’s name, year of publication, country of origin, source of control groups, genotyping method, total number of the case and control groups and each genotype. Considering parts of the studies supplied data concerning HBV related HCC risk, we collected them for a subgroup analysis.

### False-positive report probability analysis and trial sequential analysis

The False-positive report probability (FPRP) values at different prior probability levels for all significant findings were calculated as published reference studies [16–18]. Briefly, 0.2 was set as FPRP threshold and assigned a prior probability of 0.1 for an association with genotypes under investigation. A FPRP value <0.2 denoted a noteworthy association.

TSA was performed as described by user manual for trial sequential analysis [18]. After adopting a level of significance of 5% for type I error and of 30% for type II error, the required information size was calculated, and TSA monitoring boundaries were built [19,20].

### Statistics analysis

Hardy–Weinberg equilibrium (HWE) was calculated for control group using the Chi-square test and *P*<0.05 was considered to be significant disequilibrium. The strength of the association between the miRNA polymorphism and HCC risk was estimated by odds ratios (ORs) with 95% confidence intervals (CIs). In the absence of between-study heterogeneity for Q-statistic *I^2^* < 50%, fixed-effect model was reported to conserve statistical power, otherwise, the random-effect model was used [19,20]. Risk of publication bias across studies were assessed by Begg’s rank correlation and the Egger’s linear regression, and if *P*>0.10 was considered to be lack of publication bias [21]. Sensitivity analysis was conducted by eliminating studies one by one. All analyses were conducted using Stata software 11.0 and the results were considered statistically significant when the *P*-value was less than 0.05.

## Results

### Characteristics of the eligible studies

**Figure 1:**
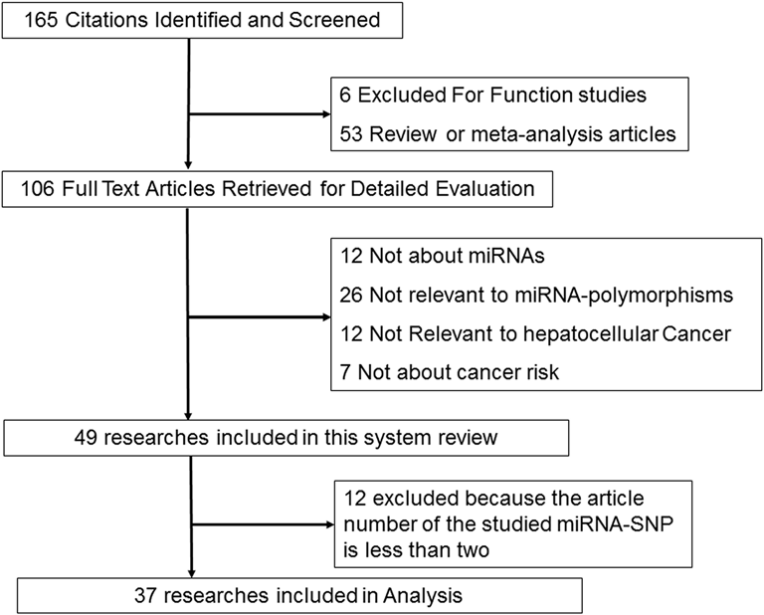
Studies identified in this meta-analysis based on the criteria for inclusion and exclusion

As shown in the flow diagram in Figure 1, a total of 165 articles were included in this systematic review, and finally, 37 researches, 11821 HCC patients and 15359 controls were involved in our meta-analysis after multiple steps of selection (Figure 1). The characteristics of each included study and the genotype frequency distributions of each SNPs are presented in Table 1. We also listed the genotype of HBV-related HCC group as data for the subgroup analysis. Then, HWE was calculated and *P* of HWE in control group for several studies did not reach genetic equilibrium, then, studies for *P*_HWE_<0.05 were excluded in the following analysis.

**Table 1:** Characteristics of literature included for this meta-analysis for HCC risk

Number	First author	Year	Country	Ethnicity	Source of control groups	Genotyping method	hsa-miRNA	Sample size		Case			Control			HBV-related HCC			*P* of HWE in control group	Citation
								Case	Control	Homozygote wild	Heterozygote	Homozygote variant	Homozygote wild	Heterozygote	Homozygote variant	Homozygote wild	Heterozygote	Homozygote variant		
1	H. Akkız	2011	Turkish	Caucasian	HB	PCR-RFLP	hsa-*mir-196a-2*	185	185	77	86	22	58	87	40	46	48	11	0.492	[49]
2	Hikmet Akkız	2011	Turkish	Caucasian	HB	PCR-RFLP	hsa-*mir-499*	222	222	45	87	90	47	93	82				0.950	[50]
3	Hikmet Akkız	2011	Turkish	Caucasian	HB	PCR-RFLP	hsa-*mir-146a*	222	222	137	75	10	144	67	11	75	51	6	0.384	[51]
4	Yin-Hung Chu	2014	China	Asian	HB	PCR-RFLP	hsa-*mir-146a*	188	337	22	82	84	50	146	141	47		32	0.230	[24]
						PCR-RFLP	hsa-*mir-196a-2*	188	337	41	81	66	70	167	100	46		33	0.986	
						PCR-RFLP	hsa-*mir-499*	188	337	119	60	9	281	55	1	46		27	0.321	
						Real-time PCR	hsa-*mir-149*	188	337	13	36	139	27	64	246	19		54	**<0.001**	
5	Ning Cong	2014	China	Asian	HB	PCR-RFLP	hsa-*mir-146a*	206	218	27	85	94	17	84	117	15	35	39	0.723	[52]
6	Yu-Xia Hao	2013	China	Asian	HB	PCR-RFLP	hsa-*mir-146a*	226	281	23	133	70	30	154	97				0.056	[53]
							hsa-*mir-196a-2*	235	282	77	126	32	67	160	55	46	71	16	0.051	
							hsa-*mir-499*	235	281	160	51	24	204	61	16				**<0.001**	
7	Won Hee Kim	2012	Korea	Asian	PB	PCR-RFLP	hsa-*mir-146a*	159	201	14	88	57	24	103	74	13	71	43	0.190	[54]
							hsa-*mir-196a-2*	159	201	34	84	41	45	107	49	24	70	33	0.356	
							hsa-*mir-499*	159	201	109	47	3	120	74	*7*	91	34	2	0.278	
							hsa-*mir-149*	159	201	14	64	81	21	97	83	68	49	10	0.345	
8	Jian-Tao Kou	2014	China	Asian	HB	PCR-RFLP	hsa-*mir-146a*	271	532	25	147	99	56	297	179				**<0.001**	[25]
							hsa-*mir-196a-2*	271	532	84	150	37	125	304	103	56	85	18	**<0.001**	
							hsa-*mir-499*	271	532	210	49	12	391	110	31				**<0.001**	
							hsa-*mir-149*	270	532	113	122	35	202	253	77				0.877	
9	D. Li	2015	China	Asian	HB	PCR-RFLP	hsa-*mir-146a*	184	184	43	83	58	52	85	47	97 (allele)		101 (allele)	0.210	[55]
							hsa-*mir-499*	184	184	128	39	17	117	43	24	146 (allele)		52 (allele)	0.780	
10	Juan Li	2016	China	Asian	NM	Sequencing	hsa-*mir-196a-2*	109	105	25	64	20	18	52	35				0.861	[56]
11	Xinhong Li	2015	China	Asian	HB	PCR-RFLP	hsa-*mir-146a*	266	266	151	86	29	166	81	19				0.060	[57]
							hsa-*mir-196a-2*	266	266	84	131	51	113	123	30	33		77	0.689	
							hsa-*mir-499*	266	266	150	92	24	166	83	17				0.140	
							hsa-*mir-149*	266	266	91	130	45	108	124	34				0.864	
12	Xiaodong Li	2010	China	Asian	HB	PCR-RFLP	hsa-*mir-196a-2*	310	222	78	150	82	42	102	78				0.402	[58]
13	M.F. Liu	2014	China	Asian	NM	Sequenom	hsa-*mir-149*	327	327	84	143	100	56	138	133	109		23	0.054	[59]
14	Y.F. Shan	2013	China	Asian	HB	PCR-RFLP	hsa-*mir-146a*	172	185	28	62	82	36	71	78	13	25	33	0.080	[60]
							hsa-*mir-499*	172	185	128	37	7	123	48	14	54	14	3	0.120	
15	Eman A. Toraih	2016	Egypt	Caucasian	PB	Real-time PCR	hsa-*mir-196a-2*	60	150	25	32	3	80	53	17				0.082	[61]
							hsa-*mir-499*	60	150	28	23	9	57	66	27				0.307	
16	X.H. Wang	2014	China	Asian	HB	PCR-RFLP	hsa-*mir-499*	152	304	98	32	22	218	62	24	59	18	12	**<0.001a**	[62]
							hsa-*mir-149*	152	304	13	72	67	43	148	113	40	42	7	0.623	
17	Yu Xiang	2012	China	Asian	HB	PCR-RFLP	hsa-*mir-146a*	100	100	27	45	28	21	46	33	18	34	21	0.506	[63]
							hsa-*mir-499*	100	100	36	40	24	54	36	10	27	30	16	0.284	
18	Teng Xu	2008	China	Asian	HB	PCR-RFLP	hsa-*mir-146a*	479	504	80	241	158	58	249	197				0.119	[64]
19	Pingping Yan	2015	China	Asian	HB	PCR-RFLP	hsa-*mir-146a*	274	328	35	145	94	36	169	123				0.050	[65]
							hsa-*mir-196a-2*	274	328	46	147	81	27	165	136	46	81	41	**0.018a**	
							hsa-*mir-499*	274	328	147	98	29	188	112	28				0.060	
							hsa-*mir-149*	274	328	66	133	75	72	156	100				0.449	
20	Jun Zhang	2013	China	Asian	PB	Sequenom	hsa-*mir-146a*	997	998	163	503	331	156	475	367	124	390	257	0.911	[66]
							hsa-*mir-196a-2*	996	995	214	488	294	165	502	328	171	376	224	0.245	
21	L.H. Zhang	2016	China	Asian	HB	PCR-RFLP	hsa-*mir-146a*	175	302	37	86	52	30	135	137				0.697	[67]
							hsa-*mir-196a-2*	175	302	25	85	65	42	138	122				0.766	
							hsa-*mir-499*	175	302	115	49	11	197	87	18				0.052	
22	Xin-wei Zhang	2011	China	Asian	PB	PIRA-PCR	hsa-*mir-146a*	925	840	156	450	319	151	386	303				0.149	[68]
							hsa-*mir-196a-2*	934	837	208	449	277	181	417	239				0.972	
23	Bing Zhou	2014	China	Asian	NM	Sequenom	hsa-*mir-146a*	266	281	40	153	73	30	154	97	24	89	40	**0.007a**	[69]
							hsa-*mir-196a-2*	266	281	93	139	34	66	160	55	57	80	16	**0.019b**	
							hsa-*mir-499*	266	281	184	59	23	204	61	16				**<0.001a**	
24	Juan Zhou	2012	China	Asian	NM	PCR-RFLP	hsa-*mir-146a*	186	483	33	86	67	71	254	158				0.056	[70]
							hsa-*mir-499*	186	483	141	41	4	371	100	12				0.100	
25	Hong-Zhi Zou	2013	China	Asian	HB	PCR-RFLP	hsa-*mir-499*	185	204	136	44	5	139	52	13	54	14	3	0.060	[71]
26	Xi-Dai Long	2016	China	Asian	HB	Real-time PCR	hsa-*mir-146a*	1706	2270	464	858	384	639	1187	444				**0.011c**	[46]
							hsa-*mir-196a-2*	1704	2270	484	867	353	718	1138	414				0.318	
							hsa-*mir-499*	1706	2270	1073	492	141	1460	598	212				**<0.001c**	
							hsa-*mir-149*	1706	2270	1104	395	207	1503	512	255				**<0.001c**	
27	Rui Wang	2014	China	Asian	PB	Sequenom	hsa-*mir-149*	172	267	21	68	83	36	105	126	16	50	57	0.066	[72]
28	Jia-Hui Qi	2014	China	Asian	PB	HRM-PCR	hsa-*mir-146a*	314	406	0	165	149	3	244	159				**<0.001a**	[73]
							hsa-*mir-196a-2*	314	406	45	209	60	71	214	121				0.156	
							hsa-*mir-499*	314	406	195	117	2	301	101	4				0.157	
29	Yanyun Ma	2014	China	Asian	HB	Sequenom	hsa-*mir-499*	981	969	724	241	16	765	179	25	558	189	13	**<0.001b**	[74]
30	Yifang Han	2013	China	Asian	PB and HB mixed	qPCR	hsa-*mir-34b/c*	1013	999	451	444	118	456	424	119				0.183	[22]
						qPCR	hsa-*mir-196a-2*	1017	1009	207	505	305	220	485	304				0.310	[75]
31	Myung Su Son	2013	Korea	Asian	HB	PCR-RFLP	hsa-*mir-34b/c*	157	201	69	75	13	110	74	17				0.371	
32	Yan Xu	2011	China	Asian	PB	PCR-RFLP	hsa-*mir-34b/c*	502	549	204	236	62	266	229	54				0.647	[36]
33	L.L. Chen	2016	China	Asian	HB	PCR-RFLP	hsa-*mir-34b/c*	286	572	102	146	38	272	267	33				**0.002a**	[76]
34	Pornpitra Pratedrat	2015	Thailand	Asian	PB	Real-time PCR	hsa-*mir-101-1*	104	95	37	51	16	39	43	13				0.835	[77]
							hsa-*mir-149*	104	95	11	27	66	9	24	62				**0.010c**	
35	Olfat Shaker	2017	Egypt	Caucasian	NM	Real-time PCR	hsa-*mir-101-1*	36	32	14	12	10	11	20	1				**0.029c**	[78]
36	Z.Y. Sui	2016	China	Asian	HB	Sequencing	let-7i	89	95	25	64		55		40				0.482	[79]
37	Fang Huang	2011	China	Asian	HB	qPCR	let-7i	1261	1319	542	564	155	581	585	153				0.756	[80]

Abbreviations: HB, hospital based; HRM-PCR, high resolution melting-PCR; NM, not mentioned; PB, population based; PCR-RFLP, PCR-restriction fragment length polymorphism; PIRA-PCR, primer introduced restriction analysis–PCR.

qPCR, quantitative polymerase chain reaction. The bold values used in ‘P of HWE in control group’ means studies did not reach genetic equilibrium and were excluded in the following analysis.

### Quantitative data synthesis of miRNA SNPs

**Figure 2:**
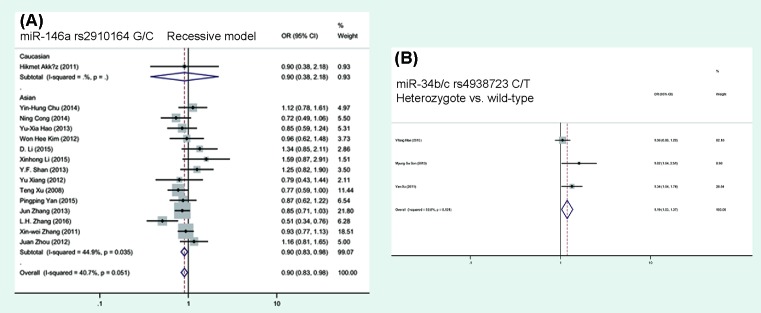
Forest plot of ORs for the association of hsa-*mir-146a* and hsa-*mir-34b/c* polymorphism with HCC risks (**A**) hsa-*mir-146a* polymorphism stratified by ethnicity in recessive model; (**B**) hsa-*mir-34b/c* polymorphism in co-dominant model (heterozygote compared with wild-type).

We found hsa-*mir-146a* rs2910164 was associated with a decreased HCC risk in the recessive model (*P*=0.017, OR = 0.90, 95%CI = 0.83–0.98; Table 2 and Figure 2). While hsa-*mir-34b/c* rs4938723 was related with an increased HCC risk in the co-dominante model (*P*=0.016, OR = 1.19, 95%CI = 1.03–1.37). In the stratified analysis, individuals carrying hsa-*mir-146a* rs2910164 variant genotype were associated with a decreased HCC risk in the Asian population subgroup (*P*=0.017, OR = 0.90, 95%CI = 0.83–0.98) while individuals carrying hsa-*mir-196a-2* rs11614913 variant genotype were related with a decreased HCC risk in the Caucasian population subgroup (*P*=0.005, OR = 0.44, 95%CI = 0.25–0.78).

**Table 2:** Meta-analysis of the association between common SNPs and HCC risk

Stratification	*n*	Heterozygote compared with wild-type		Mutation homozygote compared with wild-type			Dominant model			Recessive model			Allelic model			
		OR (95%CI)	*P*	*I^2^* (%)	OR (95%CI)	*P*	*I^2^* (%)	OR (95%CI)	*P*	*I^2^* (%)	OR (95%CI)	*P*	I^2^ (%)	OR (95%CI)	*P*	*I^2^* (%)
hsa-*mir-146a*	15	0.98	0.812	20.4	0.90	0.297	59.4^1^	0.94	0.472	50.0^1^	**0.90**	**0.017**	40.7	1.05	0.315	61.2^1^
rs2910164 G/C		(0.88–1.10)			(0.73–1.10)			(0.80–1.11)			**(0.83–0.98)**			(0.95–1.16)		
Asians	14	0.97	0.636	22.4	0.89	0.306	62.3^1^	0.93	0.383	52.1^1^	**0.90**	**0.017**	44.9	1.06	0.272	63.2^1^
		(0.87–1.09)			(0.71–1.11)			(0.78–1.10)			**(0.83–0.98)**			(0.96–1.18)		
Caucasian	1	1.18	0.430	NA	0.96	0.920	NA	1.45	0.491	NA	0.91	0.823	NA	0.92	0.619	NA
		(0.79–1.76)			(0.39–2.32)			(0.78–1.69)			(0.38–2.18)			(0.67–1.27)		
hsa-*mir-196a-2*	14	1.00	0.992	53.4^1^	0.86	0.179	73.5^1^	0.96	0.636	64.9^1^	0.88	0.122	72.1^1^	1.06	0.244	74.0^1^
rs11614913 C/T		(0.87–1.15)			(0.70–1.07)			(0.83–1.12)			(0.74–1.04)			(0.96–1.18)		
Asians	12	0.99	0.929	50.2^1^	0.92	0.420	73.2^1^	0.97	0.703	63.9^1^	0.92	0.305	72.0^1^	1.05	0.400	74.1^1^
		(0.87–1.14)			(0.70–1.07)			(0.83–1.13)			(0.78–1.08)			(0.94–1.16)		
Caucasian	2	1.17	0.743	82.8^1^	**0.44**	**0.005**	0.0	0.99	0.976	83.0^1^	**0.47**	**0.005**	0.0	1.19	0.517	73.8^1^
		(0.46–2.97)			**(0.25–0.78)**			(0.40–2.42)			**(0.28–0.79)**			(0.70–2.02)		
hsa-*mir-499*	13	1.10	0.376	67.4^1^	1.04	0.850	58.3^1^	1.11	0.410	76.7^1^	1.04	0.829	48.6^3^	0.92	0.418	81.0^1^
rs3746444 A/G		(0.89–1.37)			(0.71–1.51)			(0.87–1.40)			(0.75–1.43)			(0.74–1.13)		
Asians	11	1.14	0.264	70.7^1^	1.07	0.779	63.9^1^	1.15	0.315	79.4^1^	1.04	0.861	56.0^1^	0.89	0.367	83.4^1^
		(0.90–1.45)			(0.67–1.71)			(0.88–1.40)			(0.68–1.57)			(0.70–1.14)		
Caucasian	2	0.87	0.448	0.0	1.00	0.993	2.5	0.91	0.613	11.1	1.09	0.632	0.0	1.000	1.000	41.1
		(0.58–1.29)			(0.65–1.55)			(0.63–1.31)			(0.77–1.54)			(0.80–1.26)		
hsa-*mir-149*	7	0.97	0.696	16.6	1.03	0.882	68.2^1^	0.99	0.962	56.6^1^	1.03	0.828	61.1^1^	1.02	0.670	73.4^1^
rs2292832 C/T		(0.82–1.14)			(0.72–1.47)			(0.77–1.28)			(0.81–1.30)			(0.93–1.12)		
hsa-*mir-34b/c*	3	**1.19**	**0.016**	52.6^2^	1.15	0.221	20.4	1.25	0.065	58.6^1^	1.06	0.580	0.0	0.87	0.100	54.2^1^
rs4938723 T/C		**(1.03–1.37)**			(0.92–1.44)			(0.99–1.58)			(0.86–1.31)			(0.74–1.03)		

The results were in bold, if *P*<0.05. ^1^, means the heterogeneity exists and random-effect model based on DerSimonian and Laird method was used, otherwise, a fixed-effect model based on the Mantel–Haenszel method was employed. ^2^, *P*_heterogeneity_ is 0.121 which is higher than 0.10, thus fixed model is used. ^3^, *P*_heterogeneity_ is 0.025 which is lower than 0.10, thus random model is used.

**Figure 3:**
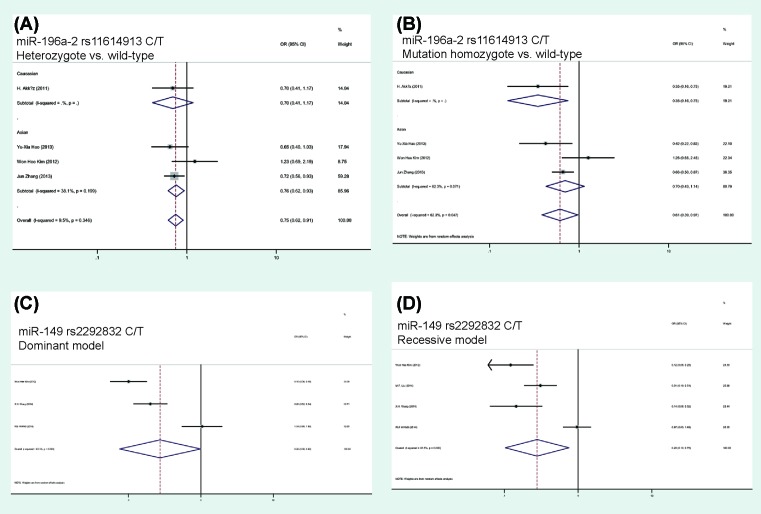
Forest plot of ORs for the association of hsa-*mir-196a-2* and hsa-*mir-149* polymorphism with HCC risks (**A**) hsa-*mir-196a-2* polymorphism stratified by ethnicity in co-dominant model (heterozygote compared with wild-type); (**B**) hsa-*mir-196a-2* polymorphism stratified by ethnicity in co-dominant model (mutation homozygote compared with wild-type); (**C**) hsa*-mir-149* polymorphism in dominant model; (**D**) hsa-*mir-149* polymorphism in recessive model.

When analyzing the HBV-related HCC risk, we found that hsa-*mir-196a-2* rs11614913 was associated with a decreased HBV-related HCC risk in the co-dominant and allelic models (CT compared with CC: *P*=0.003, OR = 0.75, 95%CI = 0.62–0.91; TT compared with CC: *P*=0.036, OR = 0.61, 95%CI = 0.39–0.97; T compared with C: *P*=0.031, OR = 0.80, 95%CI = 0.65–0.98). And hsa-*mir-149* rs2292832 was found to be associated with a decreased HBV-related HCC risk in the dominant and recessive models (dominant: *P*=0.049, OR = 0.28, 95%CI = 0.08–0.99; recessive: *P*=0.012, OR = 0.28, 95%CI = 0.10–0.75, Table 3 and Figure 3).

**Table 3:** Meta-analysis of the association between common SNPs and HBV related-HCC risk

Stratification	*n*	Heterozygote compared with wild-type			*n*	Mutation homozygote compared with wild-type			*n*	Dominant model			*n*	Recessive model			*n*	Allelic model		
		OR (95%CI)	*P*	*I^2^* (%)		OR (95%CI)	*P*	*I^2^* (%)		OR (95%CI)	*P*	*I^2^* (%)		OR (95%CI)	*P*	*I^2^* (%)		OR (95%CI)	*P*	*I^2^* (%)
hsa-*mir-146a*	6	1.05	0.627	21.9	6	0.86	0.178	8.8	6	0.99	0.950	39.2	7	0.87	0.066	0.0	7	0.95	0.281	26.3
rs2910164 G/C		(0.86–1.28)				(0.69–1.07)				(0.82–1.20)				(0.75–1.01)				(0.86–1.05)		
Asians	5	0.97	0.813	0.0	5	0.85	0.161	24.9	5	0.92	0.434	24.4	6	0.87	0.067	0.0	6	0.93	0.144	12.6
		(0.78–1.22)				(0.68–1.07)				(0.75–1.13)				(0.75–1.01)				(0.83–1.03)		
Caucasian	1	1.46	0.105	NA	1	1.05	0.930	NA	1	1.40	0.132	NA	1	0.91	0.862	NA	1	1.25	0.232	NA
		(0.92–2.31)				(0.37–2.94)				(0.90–2.18)				(0.33–2.53)				(0.87–1.80)		
hsa-*mir-196a-2*	4	**0.75**	**0.003**	9.5	4	**0.61**	**0.036**	62.3^1^	5	0.86	0.444	76.4^1^	5	0.86	0.429	70.5^1^	4	**0.80**	**0.031**	60.4^1^
rs11614913 C/T		**(0.62–0.91)**				**(0.39–0.97)**				(0.58–1.27)				(0.58–1.26)				**(0.65–0.98)**		
Asians	3	**0.76**	**0.009**	38.1	3	0.70	0.153	62.3^1^	4	0.94	0.805	80.5^1^	4	0.97	0.861	68.5^1^	3	0.85	0.130	58.0^1^
		**(0.62–0.93)**				(0.43–1.14)				(0.59–1.50)				(0.66–1.42)				(0.68–1.05)		
Caucasian	1	0.70	0.174	NA	1	**0.35**	**0.007**	NA	1	**0.59**	**0.034**	NA	1	**0.42**	**0.019**	NA	1	**0.61**	**0.006**	NA
		(0.41–1.17)				**(0.16–0.75)**				**(0.36–0.96)**				**(0.21–0.87)**				**(0.43–0.87)**		
hsa-*mir-499*	4	0.81	0.351	52.4^1^	4	0.85	0.769	68.1^1^	5	1.08	0.833	85.6^1^	4	0.90	0.818	55.5^1^	5	0.90	0.633	76.1^1^
rs3746444 A/G		(0.52–1.27)				(0.28–2.56)				(0.55–2.12)				(0.36–2.24)				(0.59–1.38)		
hsa-mir-149	3	0.37	0.059	88.7^1^	3	0.14	0.071	95.6^1^	3	**0.28**	**0.049**	93.3^1^	4	**0.28**	**0.012**	91.5^1^	3	0.38	0.057	96.0^1^
rs2292832 C/T		(0.13–1.04)				(0.02–1.18)				**(0.08–0.99)**				**(0.10-0.75)**				(0.14–1.03)		

The results were in bold, if *P*<0.05. ^1^, means the heterogeneity exists and random-effect model based on DerSimonian and Laird method was used, otherwise, a fixed-effect model based on the Mantel–Haenszel method was employed.

### Other miRNA SNPs and HCC risk

The association of some polymorphisms with HCC risk could not be evaluated because of the limited number of studies (such as hsa-*mir-101-1* rs7536540 and hsa-let-7i rs10877887). We reviewed these miRNA SNPs that have been studied for HCC cancer risk (Table 4). These may prove informative in the future study of HCC-associated miRNA polymorphism biomarkers.

**Table 4:** Other SNPs conferring in the studies of HCC risk

Number	hsa-mirNA	SNP	Results	Citation
1	hsa-*mir-646*	rs6513497	The variant allele decreased HCC risk	[81]
2	hsa-*mir-122*	rs4309483	The variant allele increased HCC risk in HBV carriers	[48]
3	hsa-*mir-378*	rs1076064	The variant allele decreased HCC risk in HBV carriers	[82]
4	hsa-*mir-501*	rs112489955	The variant allele decreased HCC risk	[47]
5	hsa-*mir-608*	rs4919510	No association	[72]
6	hsa-*mirNA3152*	rs13299349	The variant allele increased HCC risk	[83]
7	hsa-*mirNA449b*	rs10061133	The variant allele increased HCC risk	[83]
8	hsa-*mir-106b-25*	rs999885	The variant genotype increased HCC risk in HBV persistent carriers	[84]
9	hsa-*mir-199a*	rs74723057	No association	[85]
10	hsa-*mir-301b*	rs384262	No association	[73]
11	hsa-*mir-423*	rs6505162	No association	[74]
12	hsa-*mir-221*	rs17084733	No association	[78]
13	hsa-*mir-1269a*	rs73239138	The variant allele increased HCC risk	[86]

### Heterogeneity

Heterogeneity between studies was observed in Table 2. Some comparisons showed slight or moderate heterogeneity between studies. We subsequently conducted sensitivity analyses by estimating sensitivity before and after removal of each study from the analysis (Supplementary Table S1). The most influencing single study was the study conducted by Han et al. [22] for hsa-*mir-34b/c* rs4938723. However, sensitivity analysis results ranged from insignificant to statistically significant for the allele comparison because the ORs (95%CI) were 0.87 (0.73–1.03) before removal of the study by Han et al. [22] and 0.79 (0.67–0.92) after removal of that study.

### Publication bias

We used Begg’s and Egger’s tests to evaluate the potential publication bias of included studies. For hsa-*mir-149* rs2292832, a significant *P*<0.05 was observed in the three genetic models (Table 5), indicating potential publication bias. As reported, this may be due to language bias, a flawed methodological design for smaller studies or a lack of publication of small trials with opposing results [9].

**Table 5:** The results of Begg’s and Egger’s tests for the publication bias

Comparison type	Begg’s test		Egger’s test	
	Z value	*P*-value	*t* value	*P*-value
hsa-*mir-146a* rs2910164 G/C				
Heterozygote compared with wild-type	−0.64	0.520	0.71	0.490
Mutation homozygote compared with wild-type	0.05	0.961	−0.47	0.648
Dominant model	−0.54	0.586	0.43	0.673
Recessive model	1.14	0.255	−1.44	0.173
Allelic model	−0.94	0.347	0.80	0.435
hsa-*mir-196a-2* rs11614913 C/T				
heterozygote compared with wild-type	0.49	0.622	0.38	0.710
mutation homozygote compared with wild-type	−1.15	0.250	1.33	0.209
Dominant model	−0.05	0.956	0.84	0.418
Recessive model	−1.04	0.298	1.30	0.216
Allelic model	0.60	0.547	−1.08	0.300
hsa-*mir-499* rs3746444 A/G				
Heterozygote compared with wild-type	−1.59	0.113	1.78	0.103
Mutation homozygote compared with wild-type	−0.73	0.464	0.17	0.865
Dominant model	−1.22	0.222	1.25	0.237
Recessive model	−0.61	0.542	0.43	0.673
Allelic model	1.22	0.222	−0.86	0.410
hsa-*mir-149* rs2292832 T/C				
Heterozygote compared with wild-type	0.75	0.453	−1.08	0.331
Mutation homozygote compared with wild-type	1.95	**0.051**	−3.08	**0.028**
Dominant model	1.05	0.293	−1.26	0.263
Recessive model	1.65	**0.099**	−2.80	**0.038**
Allelic model	−1.95	**0.051**	2.66	**0.045**
hsa-*mir-34b/c* rs4938723 T/C				
Heterozygote compared with wild-type	1.57	0.117	−1.44	0.387
Mutation homozygote compared with wild-type	0.52	0.602	−0.21	0.867
Dominant model	0.52	0.602	−0.99	0.504
Recessive model	0.52	0.602	−0.04	0.977
Allelic model	−0.52	0.602	0.63	0.641

The bold numeric means significant as <0.100.

### FPRP analyses and trial sequential analysis

We calculated the FPRP values for all observed significant findings in the overall HCC risk. With the assumption of a prior probability of 0.1, the FPRP values in the hsa-*mir-146* rs2910164 recessive model for the overall risk and the Asian subgroups, and in the hsa-*mir-196a-2* rs11614913 recessive model for the Caucasian subgroup were all <0.20, suggesting that these significant associations were noteworthy (Table 6).

**Table 6:** FPRP values for the associations between hsa-miRNA polymorphisms and HCC risk

Variables	OR (95%CI)	*P*^1^	Power^2^	Prior probability				
				0.25	0.1	0.01	0.001	0.0001
hsa-*mir-146* rs2910164								
Recessive model								
Overall	0.90 (0.83–0.98)	0.017	0.888	**0.054**	**0.147**	0.655	0.950	0.995
Asians	0.90 (0.83–0.98)	0.017	0.870	**0.055**	**0.150**	0.659	0.951	0.995
hsa-*mir-196a*-2 rs11614913								
Mutation homozygote compared with wild-type								
Caucasian	0.44 (0.25–0.78)	0.005	0.152	**0.090**	0.228	0.765	0.970	0.997
Recessive model								
Caucasian	0.47 (0.28–0.79)	0.005	0.726	**0.020**	**0.058**	0.405	0.873	0.986
hsa-*mir-34b/c* rs4938723								
Heterozygote compared with wild-type								
Overall	1.19 (1.03–1.37)	0.016	0.353	**0.120**	0.290	0.818	0.978	0.998

PB, source of controls is population-based.

1 Chi-square test was adopted to calculate the genotype frequency distributions.

2 Statistical power was calculated using the number of observations in the subgroup and the OR and *P*-values in this table.

The bold numeric values were considered significant as <0.20.

**Figure 4:**
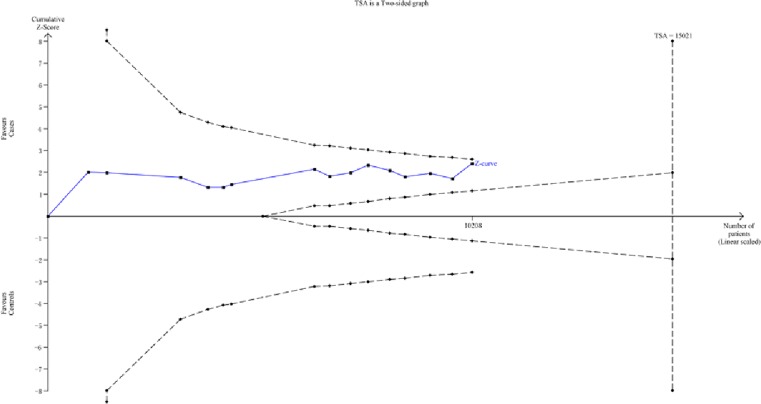
The required information size to demonstrate the relevance of hsa-*mir-146a* polymorphism with risk of HCC (recessive model)

Amongst the positive results we found, the recessive model for hsa-*mir-146a* was adopted for the trial sequential analysis to strengthen the robustness of our findings. According to TSA result, the required information size was 15021 subjects to demonstrate the issue (Figure 4). Until now, the cumulative z-curve has not crossed the trial monitoring boundary before reaching the required information size, indicating that the cumulative evidence is insufficient and further trials are necessary.

## Discussion

Until now, there was only one similar meta-analysis published [23] and we had many advantages than theirs. First, the latest update date, we searched until 23 February 2018 and there were 37 studies included in this meta-analysis. Second, we considered the available data for the HBV-related HCC risk and supplied more promising SNP sites for the precaution of HBV-related HCC risk. Third, we listed all the genotypes of the case and control groups and considered the *P*-value of HWE. There existed two problems for the research state quo: in the studying field of miRNA polymorphisms, (i) the major genotype has not the more frequencies than the minor one, which made the meta results negative. For example, hsa-*mir-149* A>G SNP was reported as 13, 36, 139 for AA, AG, GG genotype by Chu et al. [24] and as 210, 49, 12 for AA, AG, GG genotype by Kou et al. [25], while the genotyping method for them was the same. Here, we suppose the reasons for this phenomenon are the geographical and ethnicity cause and the unstable genotyping method. (ii) The Hardy–Weinberg principle was a basic law for the genetic studies. We found several studies did not mention HWE when the *P*_HWE_<0.05. In our meta-analysis, we checked the *P*-value of HWE in the control group and if *P*_HWE_<0.05, the SNP should be discarded in further analysis. In addition, we followed main directions from the guidelines for the miRNA terminology [26].

The position of miR-SNPs included pri-, pre-, and/or mature miRNA, and the function of the miR-SNPs depended on its position [27]. The pre-miR-SNPs included hsa-*mir-146a* rs2910164, hsa-*mir-196a-2* rs11614913, hsa-*mir-499* rs3746444, hsa-*mir-149* rs2292832, and hsa-*mir-27a* rs895819. Others were all pri-miR-SNPs.

In this disordered reported circumstance, we still found hsa-*mir-146a* rs2910164 and hsa-*mir-34b/c* rs4938723 had potential to be biomarkers for the HCC risk in these five common miR-SNPs. First, we found hsa-*mir-146a* rs2910164 was associated with a decreased risk of HCC. The mature hsa-*mir-146a* could function for cancer cell proliferation, apoptosis, invasion, and metastasis [28–31]. miR-SNP rs2910164 is a G to C variation located at the +4 base of the passenger strand of hsa-*mir-146a-3p*. In addition, this SNP decreases the minimum free energy (MFE) from −41.80 kcal/mol for the G allele to −38.80 kcal/mol for the C allele, suggesting a less stable secondary structure for the variant C allele. Jazdzewski et al. [32] reported that the variant (C) genotype shows lower levels of the oncogeneic hsa-*mir-146a* expression, all the above may be the reasons the variant C had a protective role for HCC risk. Second, we found that hsa-*mir-34b/c* rs4938723 was associated with an increased HCC risk. This rs4938723 located within the typical CpG island region of pri-hsa-*mir-34b/c*, and methylation of hsa*-mir-34b/c* CpG islands were reported to be associated with several cancers [33–35]. The T→C variation of this polymorphism has been predicted to create a GATA-binding site and could affect the transcription factor GATA activity and further affect the mature hsa-*mir-34b/c* expression [36], which may be the reason for the rs4938723 associated with HCC risk.

The etiology of HBV-related HCC was not caused by one particular driver mutation but involved several oncogenic pathways [37,38]. It included TP53 pathway [39], Wnt signaling [37], cell cycle [40,41], oxidative stress [39,42], epigenetic regulator [40], and so on. Thus, many miRNAs play important role for these oncogenic pathways in HBV-related HCC [43,44]. We found in this meta-analysis, hsa-*mir-196a-2* rs11614913 and hsa-*mir-149* rs2292832 were associated with decreased HBV-related HCC risks. However, there is no report about the hsa-*mir-196a-2* and hsa-*mir-149* involved in the process of HBV-related HCC. Some other miRNAs like hsa-*mir-125* were found to be associated with HBV-related HCC [45]. The results we found could be a clue for the particular miRNA involved in the pathogenic process and it also need to be verified in the future studies.

Some promising miR-SNPs were summarized in Table 5. Several SNPs were associated with HCC risk and related functional studies were also reported. For example, Long et al. [46] screened 48 pre-miRNA SNPs and found only hsa-*mir-1268a* rs28599926 affected HCC risk. And this polymorphism was associated not only with higher portal vein tumor risk and tumor dedifferentiation, but also with increasing the mutation risk of *TP53* gene and modifying the targetted *ADAMTS4* gene expression [46]. Several miR-SNPs were also found to affect the miRNA or gene expression, like hsa-*mir-501* SNP and hsa-*mir-122* SNP [47,48]. These are all the potential functional polymorphism biomarkers for the future HCC studies.

### Advantages and limitations

This meta-analysis still had several limitations. First, only studies written in English and Chinese were searched in our analysis, while reports in other languages or some other ongoing studies were not available. Second, the pooled sample size was relatively limited and thus limited for the subgroup analysis. More studies are still required to pool together to make the analysis more reliable.

### Summary and future directions

In summary, we found hsa-*mir-146a* rs2910164 was associated with a decreased HCC risk in the recessive model. While hsa-*mir-34b/c* rs4938723 was related with an increased HCC risk in the co-dominant. When analyzing the HBV-related HCC risk, hsa-*mir-196a-2* rs11614913 was associated with a decreased HBV-related HCC risk in the co-dominant and allelic models, and hsa-*mir-149* rs2292832 was found to be associated with a decreased HBV-related HCC risk in the dominant and recessive models. In conclusion, hsa-*mir-146a* rs2910164 and hsa-*mir-34b/c* rs4938723 could be biomarkers for the HCC risk while hsa-*mir-196a-2* rs11614913 and hsa-*mir-149* rs2292832 had potential to be biomarkers for HBV-related HCC risk.

## Supporting information

**Table S1:** ORs (95% CI) of sensitivity analysis.

## Abbreviations

CI: confidence interval
FPRP: false-positive report probability
HBV: Hepatitis B virus
HCC: hepatocellular cancer
HWE: Hardy–Weinberg equilibrium
OR: odds ratio
SNP: single nucleotide polymorphism
pri-miRNA: primary-microRNA
pre-miRNA: precursor-microRNA
TSA: trial sequential analysis
