# Supplementary material for: Comprehensive assessment for miRNA polymorphisms in hepatocellular cancer risk: a systematic review and meta-analysis
Source: Biosci Rep. 2018 Sep 25;38(5):BSR20180712. doi: 10.1042/BSR20180712 (PMC6153371; doi:10.1042/BSR20180712)
Supplement: Supplementary file 1 [file bsr20180712_Supp1.pdf]

Table S1. ORs (95% CI) of sensitivity analysis.

Excluding literature one by one	heterozygote vs. wild-type OR (95% CI)	mutation homozygote vs. wild-type OR (95% CI)	Dominant model OR (95% CI)	Recessive model OR (95% CI)	allelic model OR (95% CI)
miR-146a rs2910164 G/C					
Overall	0.98(0.86-1.11)	0.89(0.73-1.12)	0.94(0.80-1.11)	0.92(0.81-1.03)	1.05(0.95-1.16)
Hikmet Akkiz (2011)	0.96(0.83-1.10)	0.89(0.72-1.11)	0.93(0.78-1.10)	0.92(0.81-1.04)	1.06(0.96-1.17)
Yin-Hung Chu (2014)	0.96(0.84-1.10)	0.87(0.70-1.08)	0.92(0.78-1.09)	0.90(0.80-1.02)	1.07(0.96-1.18)
Ning Cong (2014)	0.99(0.87-1.13)	0.92(0.75-1.14)	0.97(0.82-1.13)	0.93(0.82-1.05)	1.04(0.94-1.14)
Yu-Xia Hao (2013)	0.97(0.94-1.11)	0.89(0.71-1.11)	0.94(0.79-1.11)	0.92(0.81-1.05)	1.05(0.95-1.17)
Won Hee Kim (2012)	0.96(0.84-1.10)	0.88(0.71-1.09)	0.93(0.79-1.09)	0.91(0.80-1.04)	1.06(0.95-1.17)
D. Li (2015)	0.96(0.84-1.11)	0.86(0.70-1.07)	0.92(0.78-1.09)	0.90(0.80-1.01)	1.07(0.97-1.18)
Xinhong Li (2015)	0.96(0.83-1.10)	0.86(0.70-1.06)	0.92(0.78-1.08)	0.90(0.80-1.01)	1.07(0.97-1.18)
Y.F. Shan (2013)	0.97(0.84-1.11)	0.87(0.70-1.08)	0.93(0.79-1.10)	0.90(0.80-1.10)	1.07(0.96-1.18)
Yu Xiang (2012)	0.98(0.86-1.13)	0.91(0.73-1.13)	0.95(0.81-1.13)	0.92(0.81-1.04)	1.04(0.94-1.16)
Teng Xu (2008)	1.01(0.89-1.15)	0.93(0.75-1.15)	0.98(0.84-1.15)	0.93(0.82-1.06)	1.03(0.93-1.15)
Pingping Yan (2015)	0.98(0.85-1.13)	0.90(0.72-1.13)	0.95(0.80-1.12)	0.92(0.81-1.04)	1.05(0.94-1.16)
Jun Zhang (2013)	0.97(0.83-1.12)	0.90(0.71-1.14)	0.94(0.78-1.13)	0.93(0.81-1.06)	1.05(0.94-1.17)
L.H. Zhang (2016)	1.01(0.90-1.14)	0.95(0.80-1.13)	0.99(0.87-1.13)	0.94(0.85-1.03)	1.02(0.94-1.18)
Xin-wei Zhang (2011)	0.95(0.82-1.10)	0.88(0.70-1.12)	0.93(0.78-1.11)	0.92(0.80-1.05)	1.06(0.94-1.18)
Juan Zhou (2012)	1.00(0.87-1..14)	0.89(0.71-1.12)	0.95(0.81-1.13)	0.90(0.80-1.02)	1.06(0.95-1.17)
miR-196a-2 rs11614913 C/T					
Overall	1.00(0.87-1.15)	0.86(0.70-1.07)	0.96(0.83-1.12)	0.88(0.74-1.04)	1.06(0.96-1.18)
H. Akkiz (2011)	1.02(0.88-1.18)	0.91(0.73-1.12)	0.99(0.85-1.15)	0.91(0.77-1.07)	1.04(0.94-1.15)
Yin-Hung Chu (2014)	1.01(0.88-1.17)	0.84(0.67-1.06)	0.97(0.82-1.13)	0.85(0.71-1.01)	1.06(0.96-1.20)

Yu-Xia Hao (2013)	1.03(0.90-1.18)	0.90(0.72-1.11)	1.00(0.86-1.16)	0.89(0.75-1.06)	1.04(0.94-1.16)
Won Hee Kim (2012)	1.00(0.86-1.16)	0.85(0.68-1.06)	0.96(0.82-1.12)	0.86(0.72-1.03)	1.07(0.96-1.20)
Juan Li (2016)	1.00(0.87-1.17)	0.89(0.72-1.11)	0.98(0.84-1.14)	0.91(0.77-1.07)	1.05(0.94-1.16)
Xinhong Li (2015)	0.97(0.85-1.12)	0.81(0.66-1.00)	0.93(0.80-1.07)	0.84(0.72-0.99)	1.10(0.99-1.21)
XIAO-DONG LI (2010)	1.02(0.88-1.17)	0.89(0.72-1.11)	0.99(0.85-1.15)	0.90(0.76-1.07)	1.04(0.94-1.16)
Eman A Toraih (2016)	0.98(0.85-1.12)	0.87(0.70-1.08)	0.94(0.81-1.10)	0.89(0.75-1.05)	1.07(0.96-1.19)
Jun Zhang (2013)	1.04(0.91-1.19)	0.88(0.71-1.11)	1.00(0.86-1.16)	0.87(0.73-1.05)	1.05(0.94-1.17)
L.H. Zhang (2016)	1.00(0.64-1.16)	0.86(0.68-1.08)	0.96(0.81-1.14)	0.88(0.73-1.05)	1.06(0.95-1.19)
Xin-wei Zhang (2011)	1.01(0.86-1.16)	0.84(0.66-1.08)	0.96(0.81-1.14)	0.85(0.71-1.03)	1.07(0.96-1.21)
Xi-Dai Long (2016)	0.98(0.84-1.15)	0.83(0.66-1.03)	0.94(0.80-1.11)	0.85(0.71-1.01)	1.09(0.97-1.21)
Jia-Hui Qi (2014)	0.97(0.85-1.11)	0.87(0.69-1.09)	0.95(0.81-1.11)	0.92(0.78-1.08)	1.06(0.95-1.18)
Yifang Han (2013)	0.99(0.85-1.15)	0.84(0.66-1.07)	0.95(0.80-1.12)	0.86(0.71-1.04)	1.08(0.96-1.21)
miR-499 rs3746444 A/G					
Overall	1.10(0.89-1.37)	1.04(0.71-1.51)	1.11(0.87-1.40)	1.04(0.75-1.42)	0.92(0.74-1.13)
Hikmet Akkiz (2011)	1.11(0.88-1.40)	1.02(0.66-1.58)	1.11(0.86-1.43)	1.01(0.69-1.48)	0.92(0.72-1.16)
Yin-Hung Chu (2014)	1.03(0.86-1.24)	0.97(0.70-1.35)	1.02(0.84-1.25)	1.00(0.76-1.32)	0.98(0.83-1.17)
Won Hee Kim (2012)	1.15(0.92-1.42)	1.08(0.73-1.59)	1.15(0.90-1.47)	1.07(0.77-1.48)	0.88(0.71-1.10)
D. Li (2015)	1.13(0.90-1.41)	1.09(0.73-1.63)	1.14(0.89-1.46)	1.09(0.77-1.52)	0.89(0.71-1.11)
Xinhong Li (2015)	1.09(0.86-1.38)	0.99(0.66-1.49)	1.09(0.84-1.42)	0.99(0.70-1.41)	0.93(0.73-1.17)
Y.F. Shan (2013)	1.14(0.91-1.42)	1.10(0.75-1.62)	1.15(0.90-1.47)	1.09(0.79-1.51)	0.88(0.71-1.09)
Eman A Toraih (2016)	1.13(0.91-1.41)	1.08(0.72-1.61)	1.14(0.89-1.46)	1.06(0.75-1.49)	0.89(0.71-1.12)
Yu Xiang (2012)	1.07(0.86-1.34)	0.93(0.66-1.31)	1.06(0.83-1.35)	0.95(0.71-1.28)	0.96(0.77-1.19)
Pingping Yan (2015)	1.10(0.86-1.39)	1.01(0.66-1.54)	1.10(0.84-1.43)	1.00(0.70-1.44)	0.92(0.73-1.17)
L.H. Zhang (2016)	1.11(0.88-1.41)	1.04(0.69-1.56)	1.12(0.86-1.45)	1.03(0.73-1.46)	0.91(0.72-1.15)
Juan Zhou (2012)	1.10(0.87-1.39)	1.05(0.70-1.56)	1.11(0.85-1.44)	1.05(0.74-1.47)	0.91(0.72-1.15)
Hong-Zhi Zou (2013)	1.12(0.89-1.41)	1.11(0.76-1.62)	1.14(0.89-1.46)	1.10(0.80-1.51)	0.89(0.71-1.10)

Jia-Hui Qi (2014)	1.05(0.85-1.30)	1.05(0.71-1.55)	1.06(0.83-1.35)	1.05(0.75-1.46)	0.94(0.75-1.18)
miR-149 rs2292832 C/T					
Overall	0.97(0.81-1.17)	1.03(0.72-1.47)	0.99(0.74-1.29)	1.03(0.81-1.30)	0.98(0.82-1.19)
Won Hee Kim (2012)	0.98(0.79-1.21)	0.98(0.67-1.45)	0.98(0.74-1.29)	0.97(0.76-1.23)	1.02(0.84-1.24)
JIAN-TAO KOU (2014)	1.02(0.81-1.28)	1.08(0.70-1.67)	1.04(0.76-1.43)	1.06(0.80-1.39)	0.96(0.77-1.20)
Xinhong Li (2015)	0.91(0.75-1.09)	0.95(0.65-1.38)	0.93(0.71-1.21)	0.99(0.76-1.27)	1.02(0.84-1.24)
M.F. Liu (2014)	1.03(0.86-1.24)	1.16(0.86-1.55)	1.07(0.87-1.33)	1.12(0.92-1.35)	0.92(0.80-1.07)
X.H. Wang (2014)	0.93(0.79-1.11)	0.94(0.66-1.33)	0.93(0.73-1.19)	0.98(0.76-1.27)	1.03(0.85-1.24)
Pingping Yan (2015)	0.99(0.79-1.25)	1.08(0.70-1.68)	1.03(0.74-1.31)	1.06(0.80-1.41)	0.96(0.77-1.20)
RUI WANG (2014)	0.97(0.78-1.20)	1.02(0.67-1.54)	0.98(0.74-1.31)	1.03(0.77-1.36)	0.99(0.80-1.22)
miR-34b/c rs4938723 T/C					
Overall	1.25(1.00-1.57)	1.17(0.90-1.53)	1.25(0.99-1.58)	1.06(0.86-1.31)	0.87(0.73-1.03)
Yifang Han (2013)	1.41(1.13-1.76)	1.43(1.00-2.06)	1.41(1.14-1.75)	1.22(0.86-1.72)	**0.79(0.67-0.92)**
Myung Su Son (2013)	1.17(0.93-1.48)	1.19(0.81-1.76)	1.18(0.91-1.54)	1.08(0.83-1.41)	0.89(0.72-1.10)
Yan Xu (2011)	1.25(0.83-1.86)	1.03(0.78-1.34)	1.21(0.84-1.75)	0.98(0.76-1.26)	0.91(0.74-1.13)
